# The Albumin-to-Fibrinogen Ratio Independently Predicts Acute Kidney Injury in Infants With Ventricular Septal Defect Undergoing Cardiac Surgery With Cardiopulmonary Bypass

**DOI:** 10.3389/fped.2021.682839

**Published:** 2021-07-19

**Authors:** Fan Cao, Xinxin Chen, Guodong Huang, Wenhua Liu, Na Zhou, Huili Yuan, Minghui Zou

**Affiliations:** Department of Heart Center, Guangzhou Women and Children's Medical Center, Guangzhou Medical University, Guangzhou, China

**Keywords:** ventricular septal defect, cardiopulmonary bypass, acute kidney injury, albumin-to-fibrinogen ratio, biomarker

## Abstract

**Background:** Acute kidney injury (AKI) is a common and serious complication faced by children following ventricular septal defect (VSD) surgery with cardiopulmonary bypass (CPB). The objective of this study was to explore potential predictors inherent to AKI.

**Methods:** VSD infants who were scheduled for elective cardiac surgery with CPB from 2017 to 2020 were enrolled in this study. Based on the Pediatric Risk, Injury, Failure, Loss, End-Stage Renal Disease (pRIFLE) criteria, patients were divided into AKI and non-AKI groups. Univariate and multivariate logistic regression analyses were carried out in order to evaluate potential risk factors for AKI. Receiver operating characteristic (ROC) curves were generated to evaluate the predictive probabilities of risk factors for AKI.

**Results:** Of all the 338 enrolled VSD infants, 49 manifested AKI with an incidence of 14.5% (49/338). The ROC curve indicated that albumin-to-fibrinogen ratio (AFR) during CPB was a significant predictor of AKI [area under the curve (AUC), 0.711; *p* < 0.001]. Based on the univariate and multivariate logistic analyses, AFR during CPB [odds ratio (OR), 1.89; 95% confidence interval (CI), 1.22–2.76, *p* = 0.011] was the only independent risk factor for AKI.

**Conclusions:** This study demonstrated that a low AFR (<9.35) during CPB was an independent risk factor for AKI in VSD infants following cardiac surgery with CPB.

## Introduction

Ventricular septal defect (VSD) is the most frequent congenital heart disease (CHD) as seen in both adults and children, which accounts for ~40% of all CHDs (1). Repair surgery with cardiopulmonary bypass (CPB) was a primary therapeutic strategy for infants suffering from VSD (2). CPB is a major contributor to postoperative complications and morbidity, particularly in the case of pediatric cardiac surgery (3). Acute kidney injury (AKI) is well-established as a common and serious complication seen in children following CHD surgery with CPB (4). AKI strongly affects short- and long-term outcomes, and it has been identified as an independent risk factor for a high morbidity and mortality rate in the wake of CPB (5, 6). Therefore, it is crucially important to predict AKI among VSD infants undergoing cardiac surgery with CPB.

The presence of diabetes (7), chronic kidney disease (8), sepsis and septic shock (9), and nephrotoxic agents (10) are independent risk factors with respect to AKI following cardiac surgery. However, comparatively few studies have made an attempt to examined hematological biomarkers as independent predictors of AKI. Albumin (Alb), a critical liver function index, can serve as a useful indicator for inflammatory and nutritional status (11). Fibrinogen (Fib), an essential protein in coagulation, is also widely recognized as a sensitive index for inflammatory status (12). Alb-to-Fib ratio (AFR), which combines Alb and Fib, has been used as prognostic factors for different classes of diseases, including peritonitis-induced sepsis (13), advanced epithelial ovarian cancer (14), untreated chronic lymphocytic leukemia (15), and nonsmall cell lung cancer (16). However, it is unknown whether AFR is able to serve as an independent prognostic marker for AKI in VSD infants after cardiac surgery with CPB. The objective of this study was to explore potential predictors of AKI and provide novel insights into AKI prevention.

## Materials and Methods

### Patients

The retrospective study protocol was reviewed and approved by the Ethics Committee of our hospital. Infants diagnosed with VSD at the Department of Cardiovascular Center, Guangzhou Women and Children's Medical Center during the period from 2017 to 2020 were enrolled in this study. Inclusion criteria were the following: (1) aged < 1 year, (2) diagnosed with VSD and planned for elective cardiac surgery with CPB, and (3) provided written informed consent with parents' signatures. Exclusion criteria were the following: (1) with renal dysfunction prior to surgery; (2) with the conditions that impact Alb and Fib expressions, e.g., liver function disorder, coagulation/blood dysfunction, etc.; (3) with multiple operations simultaneously or within 14 days; (4) with incomplete clinical data; and (5) followed up <30 days following surgery.

### Treatment

All the enrolled infants underwent the same operation and CPB. All surgeries were performed *via* routine median sternotomy incision under CPB and cardiac incision including right atrial incision, main pulmonary artery incision, or a combination. Extracorporeal circulation was established by conventional cannulation of the ascending aorta and superior–inferior venae cava. CPB was carried out in moderate hypothermia, with the rectal temperature at 32°C. After the ascending aorta being cross clamped, histidine–tryptophan–ketoglutarate cardioplegic solution was perfused through the aortic root. After cardiac arrest, a left ventricular vent was placed through the foramen ovale, and VSD was exposed *via* the tricuspid valve. VSD repair was performed with intermittent or continuous suture using autologous pericardium as the repair material. Blood samples were harvested during CPB (30 min after CPB initiation) for the subsequent laboratory analyses. The left ventricular vent was then pulled out, and the foramen ovale was sutured. The ascending aorta was opened, and rewarming was started with the satisfied left heart venting. The right atrial incision was closed, and the patients were gradually weaned from CPB.

### Data Collection

The clinical data were collected and laid out as below: (1) demographic data, including age, gender, weight, and height; (2) preoperative clinical data, including the Risk Adjustment for Congenital Heart Surgery 1 (RACHS-1) score (17), history of pneumonia and chronic heart failure, and left ventricular ejection fraction (LVEF); (3) intraoperative data, including duration of CPB and aortic cross-clamping (ACC), ultrafiltration, hypothermic circulatory arrest, and urine output; (4) postoperative clinical and outcomes data, including duration of postoperative mechanical ventilation, postoperative hypotension, inputs of blood products, AKI, and duration of cardiac care unit (CCU) and hospital stay; and (5) laboratory variables during CPB, including hemoglobin (Hb), glucose, lactic acid, creatinine, blood urea nitrogen (BUN), total cholesterol, triglycerides, low-density lipoprotein cholesterol (LDL-C), Alb, and Fib.

### Definitions

According to the Pediatric Risk, Injury, Failure, Loss, End-Stage Renal Disease (pRIFLE) criteria (18), AKI was defined when the postoperative creatinine clearance (CC) within 48 h was 25% lower than at the preoperative level, or the urine volume <0.5 ml/(k·h) ≥ 8 h.

### Statistical Analysis

Data were analyzed using SPSS version 19.0 (SPSS Inc., Chicago, IL, USA) and GraphPad version 8.0 (GraphPad Inc., California, CA, USA). Prior to the study, we performed a simple sample size estimation: (10–15) × the number of covariates (*n* = 27 in our study) based on the guidance of the statistical textbook. Thus, the minimum required sample size was 270, and 338 infants were included in this study with a calculated power value >0.8 using GPower 3.1. Quantitative variables were presented as mean ± standard deviation (SD) while qualitative variables as number with percentage (*n*, %). Continuous variables were compared utilizing Student's *t*-test for normal distributed data or the Mann–Whitney test for non-normal distributed data. Categorical variables were compared by means of a chi-squared test or Fisher's exact test. Receiver operating characteristic (ROC) curves were generated to evaluate the predictive probabilities of risk factors for AKI using the area under the curve (AUC), 95% confidence interval (CI), specificity, and sensitivity. The optimal cutoff value was determined by calculating the Youden index of the ROC curve. Based on the cutoff, continuous data were categorized into high (≥cutoff) and low (<cutoff) expression groups. To analyze possible risk factors of AKI, we applied a binary univariate logistic regression, followed by multivariate logistic regression with the “Enter” method. Only those factors with a *p* < 0.05 in the univariate logistic model were included in the multivariate logistic model. A two-tailed *p* < 0.05 was considered statistically different.

## Results

**Figure 1:**
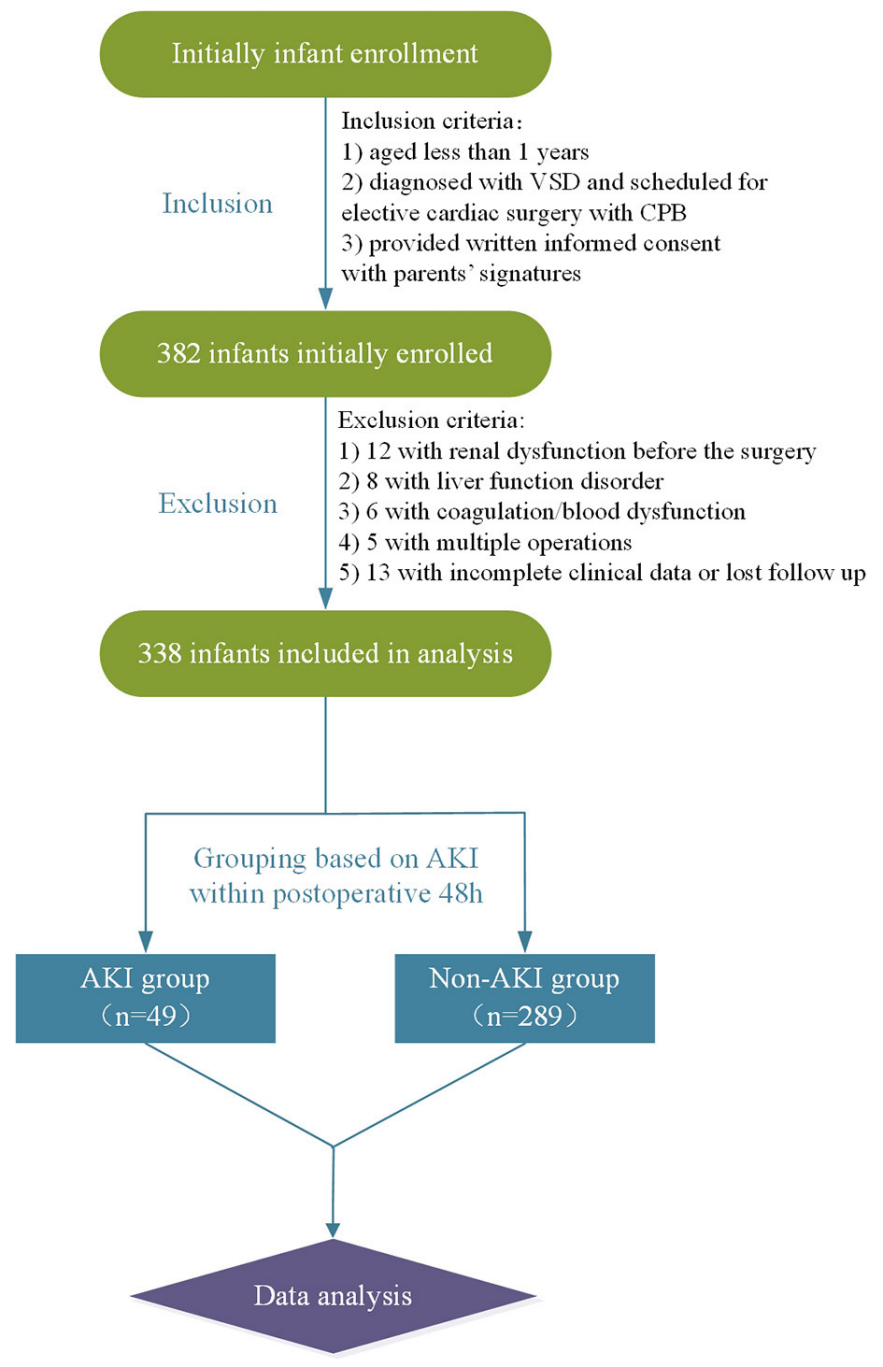
Flow chart. VSD, ventricular septal defect; CPB, cardiopulmonary bypass; AKI, acute kidney injury.

As demonstrated by the flow chart diagram seen in Figure 1, 382 VSD infants were initially enrolled, and 44 were then excluded according to the exclusion criteria. As a result, 338 infants were finally included in the data analysis. Of all the enrolled patients, 49 exhibited AKI with an incidence of 14.5% (49/338). The overall mean age of was 6.5 months (median, 6 months), and the majority were male (55.3%, 187/338). The demographic, preoperative, intraoperative, and postoperative clinical and outcomes data associated with AKI development are summarized in Table 1. The rate of RACHS-1 score >2 was significantly higher for patients with AKI than those without (51.0 vs. 29.1%, *p* = 0.002). Those infants with a longer duration of CPB and ACC were associated with an increased risk of AKI (*p* = 0.009 and 0.026, respectively). Patients with the presence of preoperative chronic heart failure (*p* = 0.025), hypothermic circulatory arrest (*p* = 0.037), and a lower preoperative LVEF (*p* = 0.004) were more likely to develop AKI. Moreover, AKI patients were associated with a prolonged CCU (*p* = 0.001) and hospital stay (*p* < 0.001). However, no significant differences were observed in terms of age, gender, weight, height, presence of pneumonia, duration of postoperative mechanical ventilation, ultrafiltration, postoperative hypotension, urine output, and the inputs of blood products between AKI and non-AKI groups (*p* > 0.05).

**Table 1:** Clinical parameters associated with AKI in VSD infants after cardiac surgery with CPB.

	**AKI**		
**Parameters**	**Yes (*n* = 49)**	**No (*n* = 289)**	***p*-value**
Age (months)	6.2 ± 1.6	6.5 ± 2.1	0.341
Gender, *n* (%)	–	–	0.973
Male	27 (55.1)	160 (55.4)	–
Female	22 (44.9)	129 (44.6)	–
Weight (kg)	3.9 ± 0.7	4.0 ± 0.6	0.294
Height (cm)	54.4 ± 3.2	54.8 ± 3.5	0.455
RACHS-1 score	–	–	0.002^*^
≤ 2	24 (49.0)	205 (70.9)	–
>2	25 (51.0)	84 (29.1)	–
History of diseases, *n* (%)	–	–	–
Pneumonia	4 (8.2)	15 (5.2)	0.169
Chronic heart failure	9 (18.4)	26 (9.0)	0.025^*^
LVEF (%)	52.3 ± 2.3	53.5 ± 2.7	0.004^*^
Duration of CPB (min)	75.4 ± 26.7	65.3 ± 24.5	0.009^*^
Duration of ACC (min)	41.3 ± 15.4	36.5 ± 13.6	0.026^*^
Duration of postoperative mechanical ventilation (h)	46.3 ± 33.2	42.4 ± 30.8	0.418
Ultrafiltration, *n* (%)	42 (85.7)	234 (81.0)	0.427
Hypothermic circulatory arrest, *n* (%)	9 (18.4)	25 (8.7)	0.037^*^
Postoperative hypotension, *n* (%)	13 (26.5)	52 (18.0)	0.161
Urine output (ml/kg/h)	1.68 ± 0.36	1.70 ± 0.39	0.738
Inputs of blood products (ml)	595.4 ± 202.5	610.4 ± 192.3	0.617
Duration of CCU stay (days)	5.0 ± 2.5	3.8 ± 2.3	0.001^*^
Postoperative hospital stay (days)	11.2 ± 4.5	8.9 ± 3.9	<0.001^*^

*AKI, acute kidney injury; VSD, ventricular septal defect; RACHS-1, Risk Adjustment for Congenital Heart Surgery 1; LVEF, left ventricular ejection fraction; CPB, cardiopulmonary bypass; ACC, aortic cross-clamping; CCU, cardiac care unit*.

*p-values were calculated by Student's t-test, chi-square test, or Fisher's exact test*.

* *p < 0.05*.

Table 2 compares the laboratory variables between patients with or without AKI. No difference was observed with respect to Hb, BUN, total cholesterol, triglycerides, and LDL-C. Patients with AKI displayed remarkably higher levels of glucose (*p* = 0.017), lactic acid (*p* = 0.020), and creatinine (*p* = 0.002) during CPB when compared with those without AKI. The AFR level in the AKI group was notably lower than that of the non-AKI group (8.5 ± 1.9 vs. 10.4 ± 2.1, *p* < 0.001).

**Table 2:** Laboratory tests associated with AKI in VSD infants after cardiac surgery with CPB.

	**AKI**		
**Laboratory tests during CPB**	**Yes (*n* = 49)**	**No (*n* = 289)**	***p*-value**
Hb (g/L)	115.4 ± 9.8	117.2 ± 10.5	0.264
Glucose (mmol/L)	9.3 ± 1.6	8.8 ± 1.3	0.017^*^
Lactic acid (mmol/L)	2.2 ± 0.8	2.0 ± 0.5	0.020^*^
Creatinine (μmol/L)	79.1 ± 12.4	73.5 ± 11.2	0.002^*^
BUN (mmol/L)	5.1 ± 1.2	4.9 ± 1.1	0.246
Total cholesterol (mmol/L)	4.6 ± 0.8	4.5 ± 1.0	0.507
Triglycerides (mmol/L)	1.7 ± 0.4	1.6 ± 0.5	0.185
LDL-C (mmol/L)	2.6 ± 0.7	2.5 ± 0.6	0.294
AFR	8.5 ± 1.9	10.4 ± 2.1	<0.001^*^

*AKI, acute kidney injury; VSD, ventricular septal defect; CPB, cardiopulmonary bypass; Hb, hemoglobin; BUN, blood urea nitrogen; LDL-C, low-density lipoprotein cholesterol*.

*p-values were calculated by Student's t test or Mann–Whitney U-test*.

* *p < 0.05*.

**Figure 2:**
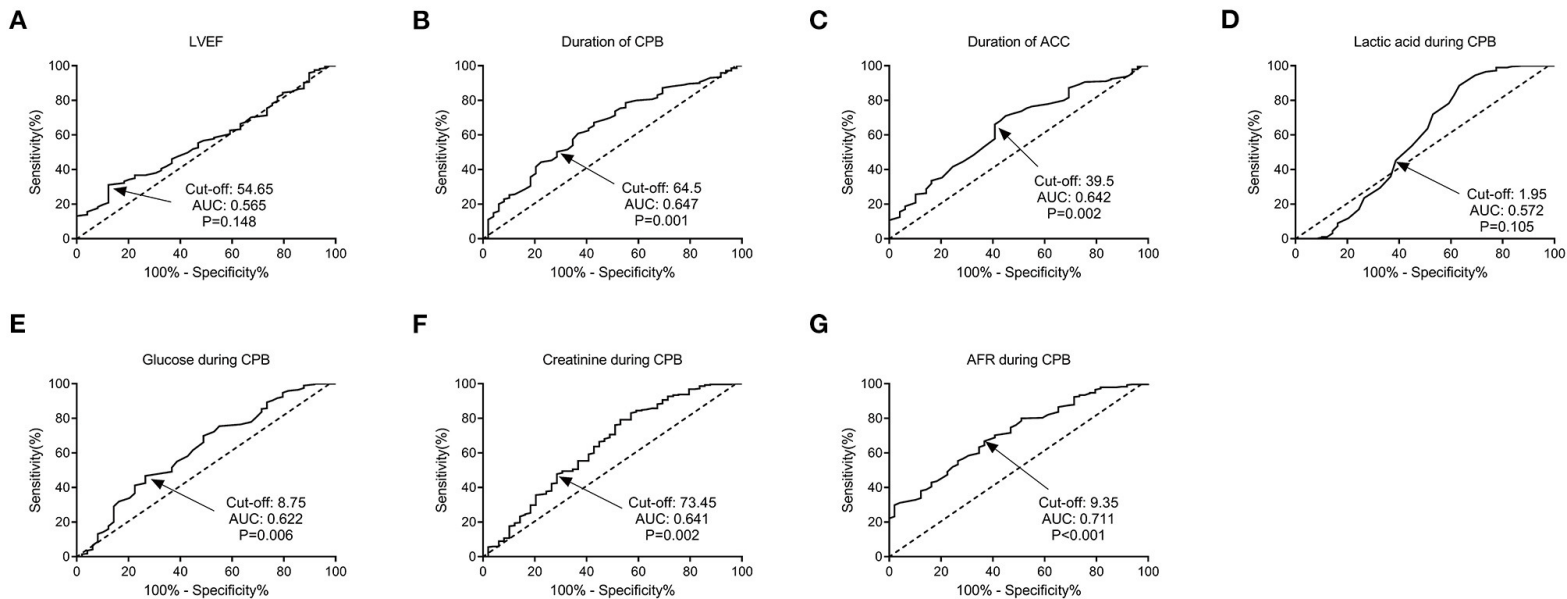
Predictive values of factors for AKI in VSD infants by ROC curve analysis. **(A)** LVEF; **(B)** duration of CPB; **(C)** duration of ACC; **(D)** lactic acid; **(E)** glucose; **(F)** creatinine; and **(G)** AFR during CPB. AFR was the most significant predictor for AKI, with a cut-off value of 9.35, an AUC of 0.711, a sensitivity of 66.78%, and a specificity of 63.27% (*P* < 0.001). VSD, ventricular septal defect; CPB, cardiopulmonary bypass; AKI, acute kidney injury; LVEF, left ventricular ejection fraction; ACC, aortic cross-clamping; AFR, albumin-to-fibrinogen ratio; ROC, receiver operating characteristic; AUC, area under the curve.

**Figure 3:**
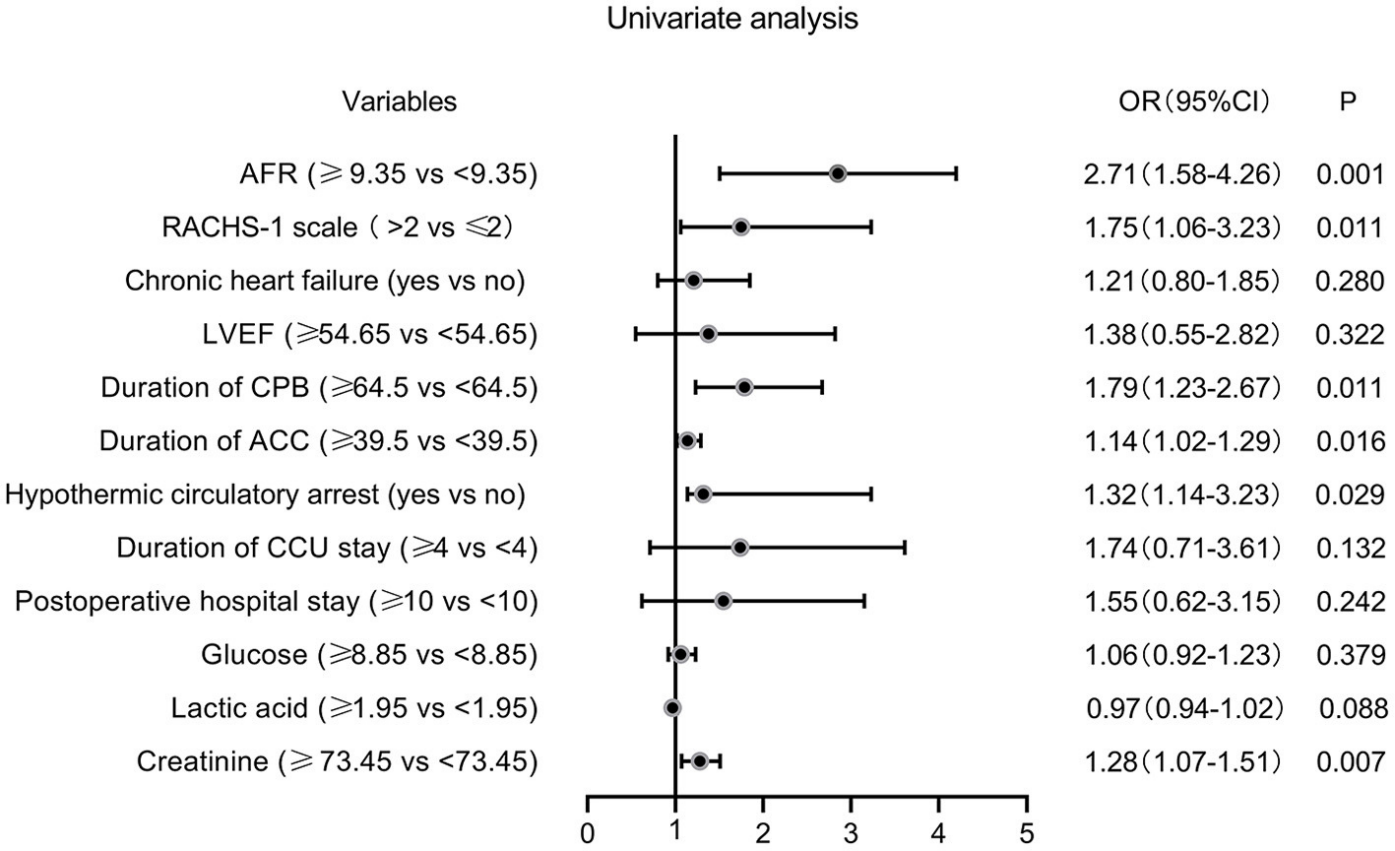
Forest plot of the univariate logistic regression analysis. AFR, albumin-to-fibrinogen ratio; RACHS-1, Risk Adjustment for Congenital Heart Surgery 1; LVEF, left ventricular ejection fraction; CPB, cardiopulmonary bypass; ACC, aortic cross-clamping; CCU, cardiac care unit; OR, odds ratio; CI, confidence interval.

**Figure 4:**
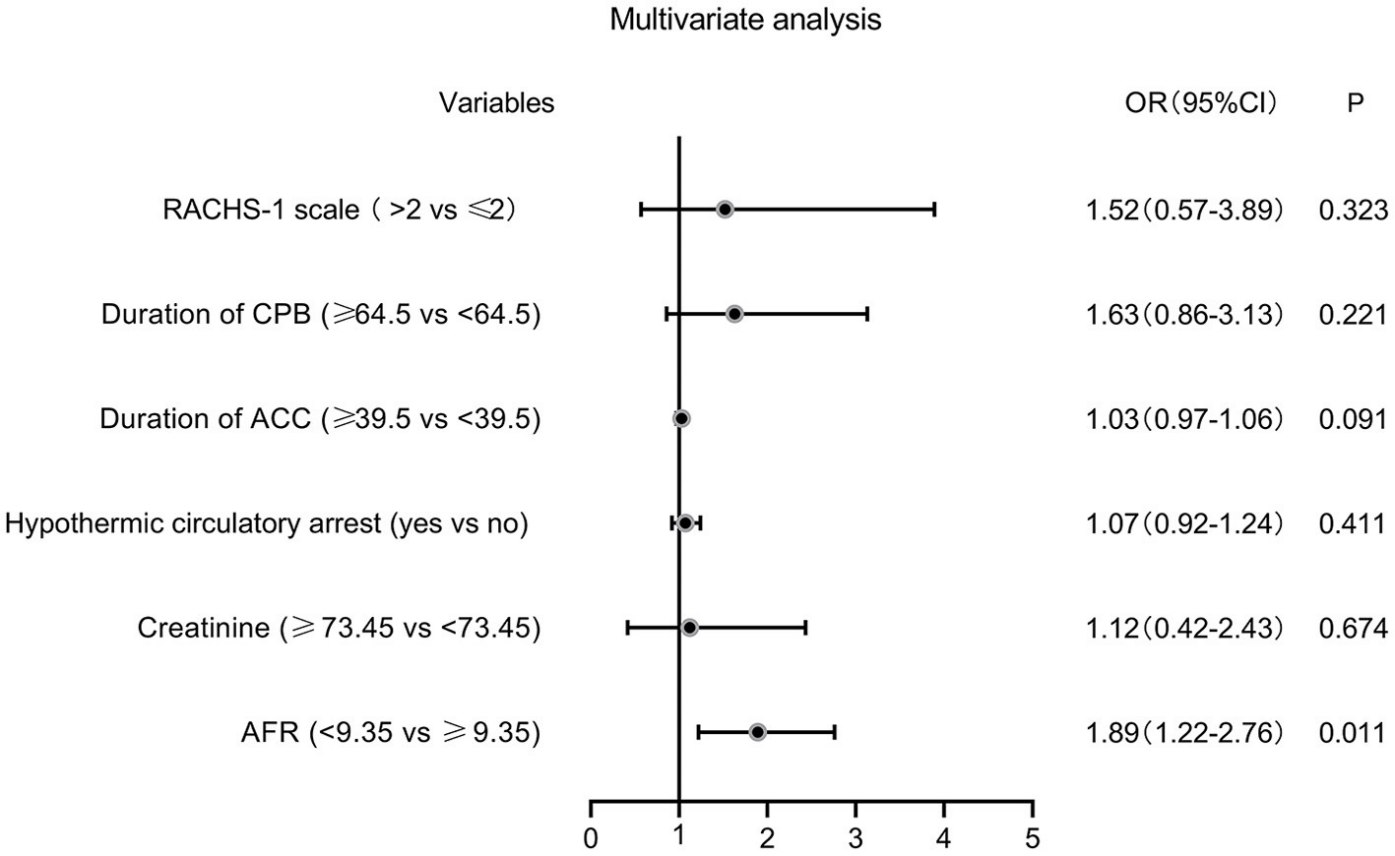
Forest plot of the multivariate logistic regression analysis. AFR, albumin-to-fibrinogen ratio; RACHS-1, Risk Adjustment for Congenital Heart Surgery 1; CPB, cardiopulmonary bypass; ACC, aortic cross-clamping; OR, odds ratio; CI, confidence interval.

The ROC curves were used to determine the predictive and cutoff values of continuous variables for AKI. As shown in Figure 2, the duration of CPB (AUC, 0.647; sensitivity, 50.17%; specificity, 71.43%; *p* = 0.001), ACC (AUC, 0.642; sensitivity, 66.09%; specificity, 59.18%; *p* = 0.002), glucose (AUC, 0.622; sensitivity, 46.71%; specificity, 73.47%; *p* = 0.006), creatinine (AUC, 0.641; sensitivity, 49.48%; specificity, 69.39%; *p* = 0.002), and AFR (AUC, 0.711; sensitivity, 66.78%; specificity, 63.27%; *p* < 0.001) were significant predictors of AKI. Based on the cutoff values, these continuous variables were categorized into two groups (high vs. low). Twelve potential risk factors (*p* < 0.05 in Tables 1, 2) were included in the univariate logistic regression model. As illustrated in Figure 3, RCAHS-1 score [odds ratio (OR), 1.89; 95% CI, 1.22–2.76; *p* = 0.011], duration of CPB (OR, 1.89; 95%CI, 1.22–2.76; *p* = 0.011), and ACC (OR, 1.89; 95% CI, 1.22–2.76, *p* = 0.011), hypothermic circulatory arrest (OR, 1.89; 95% CI, 1.22–2.76; *p* = 0.011), creatinine (OR, 1.89; 95% CI, 1.22–2.76, *p* = 0.011), and AFR (OR, 1.89; 95% CI, 1.22–2.76; *p* = 0.011) during CPB were six identifiable risk factors for AKI. These significant factors were further added to the multivariate logistic model. The forest plot in Figure 4 indicated that AFR during CPB (OR, 1.89; 95% CI, 1.22–2.76, *p* = 0.011) was the only independent risk factor pertinent to AKI in VSD infants with CPB.

## Discussion

This study indicated for the first time that a lower AFR during CPB (<9.35) was an independent risk factor for AKI occurrence in VSD infants following operation with CPB. AKI is a common and serious postoperative complication in patients after cardiac surgery (19). The incidence of AKI following pediatric cardiac surgery varies widely across different studies, ranging from 11% (20) to 52% (21). In this present study, the incidence of AKI was calculated as 14.5%, which was quite in accordance with the reported 14.1% by Lee et al. (22) and 13.2% by Fuhrman et al. (23). In our minds, different patient characteristics (e.g., age ranges, races, operation types, etc.), sample sizes, and definitions of AKI may serve to explain the various incidences of AKI in different studies. As indicated by many studies, AKI has been recognized as a risk factor for a prolonged hospitalization, a more complicated clinical course, and increased mortality following cardiac surgery (21). However, the early diagnosis of AKI is quite challenging due to its diverse clinical manifestations, varying from asymptomatic to oliguria, and renal failure. Therefore, it is of great clinical importance to investigate potential risk factors inherent to AKI. Previous reports by Rosner et al. (24) revealed that cardiac-surgery-associated characteristics, including the use of CPB, duration of CPB and ACC, and high levels of vasopressors, were closely correlated with increased risks of AKI occurrence. The altered renal perfusion, ischemia/reperfusion injury, elevated oxidative damages, and inflammation are the potential explanations for the development of AKI (25). Our univariate logistic analysis also supported the potential association between duration of CPB, ACC, and AKI development.

Alb and Fib are two widely reported proteins with inflammatory, nutritional, and hemorheological properties (26). Alb, an important serum component, is a significant regulator of colloid osmotic pressure (27). A low level of Alb is widely observed under inflammatory stimuli, due to decreased synthesis and enhanced catabolism (28). In addition, Alb can act to inhibit the activation and aggregation of platelets (29), and a decreased level will typically lead to increased blood viscosity and impaired endothelial function (30). Fib, an important coagulation factor, is a regulatory component for inflammation *via* modulating the functions of leukocyte effector and leukocyte transmigration (31). The circulated Fib level is noteworthy for being upregulated in inflammatory status, and it can promote the aggregation of platelets (32, 33). Moreover, significantly increased Fib in kidney tissues is observed following AKI (34). Alb and Fib were both reported to be significant prognostic factors in the case of a variety of diseases (35–38). AFR, an index integrating Alb and Fib, offers a more sensitive reflection of the status of systemic inflammation, blood thrombogenicity, and viscosity when compared with Alb or Fib alone (39). Mechanisms of AKI following cardiac surgery include perioperative renal ischemia, reperfusion injury, inflammation, hemolysis, and pigment nephropathy caused by CPB (25, 40). Previous studies indicate hypoalbuminemia as a modifiable risk factor for AKI in patients with off-pump coronary artery bypass surgery (25). We considered that these may be possible explanations for the predictive role of AFR for AKI occurrence.

Some limitations should be concerned. First, the sample size is not large enough. Second, this is a retrospective study that may harbor potential selection bias. Third, the laboratory variables were detected during CPB without dynamic monitoring. Lastly, whether any benefit can be derived from AFR modulation (e.g., Alb infusion) for AKI prevention remains unclear. Additional prospective multicenter studies addressing larger sample sizes and experimental studies are necessary to verify our results.

## Conclusions

In conclusion, this study demonstrated that a low AFR (<9.35) during CPB was an independent risk factor for AKI in VSD infants following cardiac surgery with CPB. In our minds, AFR evaluation will aid early prediction and secondary prevention of AKI. AFR, as an easily available biomarker, has a potential in terms of clinical practice for prognosis improvement.

## Data Availability Statement

The raw data supporting the conclusions of this article will be made available by the authors, without undue reservation.

## Ethics Statement

This retrospective study protocol was reviewed and approved by the Ethics Committee of our hospital. Written informed consent to participate in this study was provided by the participants or their legal guardian/next of kin.

## Author Contributions

FC, MZ, and GH: project development, data collection, data analysis, and manuscript writing. WL, NZ, and HY: data collection, project development. XC: project development and data analysis. All authors contributed to the article and approved the submitted version.

## Conflict of Interest

The authors declare that the research was conducted in the absence of any commercial or financial relationships that could be construed as a potential conflict of interest.
